# Workload and spirometry associated with untethered swimming in horses

**DOI:** 10.1186/s12917-024-04143-3

**Published:** 2024-07-19

**Authors:** R. Leguillette, P. McCrae, S. Massie, S. Arroyo Filho, W. Bayly, F. David

**Affiliations:** 1 Faculty of Veterinary Medicine, University of Calgary, 3330 Hospital Dr NW, Calgary, AB T2N4N1 Canada; 2 Al Shaqab, a Member of Qatar Foundation, Al Shaqab Street, Al Rayyan, Doha, Qatar; 3 Department of Veterinary Clinical Sciences, Washington State University, PO Box 646610, Pullman, WA 99164 USA; 4 EquiTom – Namur, a member of the Equine Care Group, 15 Chaussée de Nivelles, Mazy, 5032 Belgium

**Keywords:** Equine, Respiration, Ventilation, Oxygen consumption, Swimming, Workload

## Abstract

**Background:**

Swimming has been used empirically for rehabilitation and conditioning of horses. However, due to challenges imposed by recording physiological parameters in water, the intensity of free swimming effort is unknown.

**Objectives:**

Measure the physiological workload associated with untethered swimming in horses.

Five fit Arabian endurance horses were assessed while swimming in a 100 m-long indoor pool. Horses were equipped with a modified ergospirometry facemask to measure oxygen consumption (V̇O_2_) and ventilatory parameters (inspired/expired volumes, V_I_, V_E_; peak inspiratory/expiratory flows, PkV_I_, PkV_E_; respiratory frequency, Rf; minute ventilation, VE; inspiratory/expiratory durations and ratios, t_I_, t_E_, t_I_/t_tot_, t_E_/t_tot_); and an underwater electrocardiogram that recorded heart rate (HR). Postexercise venous blood lactate and ammonia concentrations were measured. Data are reported as median (interquartile ranges).

**Results:**

Horses showed bradypnea (12 breaths/min (10–16)) for the first 30 s of swimming. V̇O_2_ during swimming was 43.2 ml/(kg.min) (36.0–56.6). Ventilatory parameters were: V_I_ = 16.7 L (15.3–21.8), V_E_ = 14.7 L (12.4–18.9), PkV_I_ = 47.8 L/s (45.8–56.5), PkV_E_ = 55.8 L/s (38.3–72.5), Rf = 31.4 breaths/min (20.0–33.8), VE = 522.9 L/min (414.7–580.0), t_I_ = 0.5 s (0.5–0.6), t_E_ = 1.2 s (1.1–1.6), t_I_/t_tot_ = 0.3 (0.2–0.4), t_E_/t_tot_ = 0.7 (0.6–0.8). Expiratory flow tracings showed marked oscillations that coincided with a vibrating expiratory sound. HR was 178.0 bpm (148.5–182.0), lactate = 1.5 mmol/L (1.0–1.9) and ammonia = 41.0 µmol/L (36.5–43.5).

**Conclusions:**

Free (untethered) swimming represents a submaximal, primarily aerobic exercise in horses. The breathing pattern during swimming is unique, with a relatively longer apneic period at the beginning of the exercise and an inspiratory time less than half that of expiration.

## Background

Swimming has been used empirically for the rehabilitation and conditioning of horses for many years [1–10]. According to a recent survey of 270 Australian Standardbred racehorse trainers, 60% of leading trainers and 38% of all trainers used swimming as a form of cross-training. Among these trainers, 79% reported using swimming to replace trackwork for horses with limb injuries, and approximately 63% reported using swimming exercise to improve or maintain fitness [2].

Swimming exercise is believed to develop basic aerobic fitness; however, the intensity of this form of exercise is not well understood. In human athletes, unloaded swimming is reported to be a strenuous, though submaximal, form of exercise [11, 12]. Similarly, it has been suggested to be a submaximal exercise equivalent to jogging in Standardbred horses based on heart rate and lactate [13]. Cardiovascular responses of horses during swimming, including electrocardiograms (ECGs), have previously been described [4, 5, 14–17]. Maximum swimming heart rates (HRs) have been reported to range between approximately 145 to 216 bpm for free and tethered swimming [1, 3–5, 7, 13, 17]. Postswim (free or tethered) lactate levels range from approximately 1 to 13 mmol/L [4, 5, 7, 18, 19]. However, little information is available on the respiratory responses of horses to swimming [3–5, 14–16, 20]. The breathing pattern during swimming has previously been described as consisting of a maximum short inspiration followed by a forced end-expiratory effort [15]. In a recent study of eight race-fit horses, all the horses displayed a period of apnea upon entry into the pool. It has been postulated that this apneic period is associated with greater buoyancy or is a result of the mammalian dive reflex (MDR) [3, 20]. After this apneic period, a majority of the horses adopted the breathing strategy previously described [3]. Horses were observed to raise the upper lip and collapse the external nares [3, 15], which was accompanied by collapse of the upper respiratory tract [3]. Swimming respiratory frequencies (Rfs) are significantly lower than those observed during overground exercise, at an average of 24–28 breaths/min, with an expiratory period (T_E_) that is approximately double the inspiratory period (T_I_) [3–5, 14]. Oxygen consumption (V̇O_2_) was previously found to increase linearly with increasing work effort from 25 to 112 ml/(kg.min) in horses during tethered swimming [4]. However, no data are available for the V̇O_2_ of horses during free swimming. Therefore, the objective of this study was to assess the workload associated with free (untethered) swimming in horses. We hypothesized that untethered swimming is a low-intensity exercise with an irregular breathing pattern.

## Results

All the horses tolerated the ergospirometry facemask and swimming exercise, and no horses had to be excluded for behavioral, safety, or other issues. One horse submerged the mask for less than a second, resulting in data loss for the last 20 m of the swimming length. The horses completed the swimming length in 93 s (86–97).

The environmental conditions were as follows: ambient temperature, 31.0°C (31.0–32.0); water temperature, 31.0°C (31.0–31.0); barometric pressure, 760.0 mmHg (759.0–760.0); and humidity, 65.0% (63.0–69.0%).

### Swimming style

**Fig. 1:**
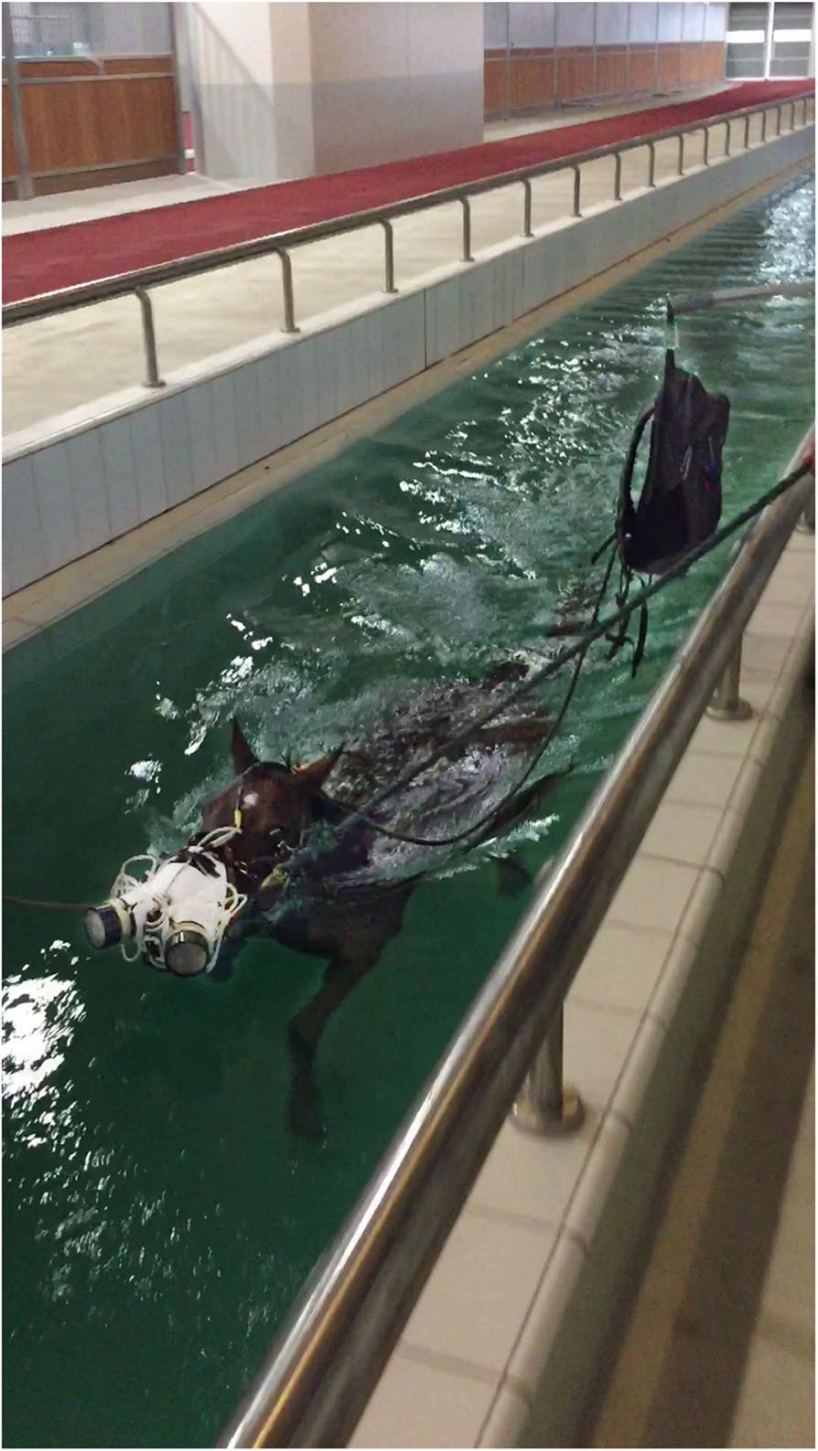
Ergospirometry facemask used during free swimming to measure oxygen consumption and ventilatory parameters

The horses were included in the study if they had been observed carrying their head above water during regular training swimming sessions. One horse swam with its head above the water more than the other horses by keeping its jaw out of contact from the water, while the other horses had only their lower lips and chin above the water (Fig. 1). One horse was keeping its croup underwater and was swimming at a slight upward body angle, whereas all the other horses had their croup submerged just slightly under water line with a horizontal body position. No unusual swimming style was observed, with all the horses moving all four legs under water and displaying a typical rotation of the hips.

### Oxygen consumption and ventilation

**Fig. 2:**
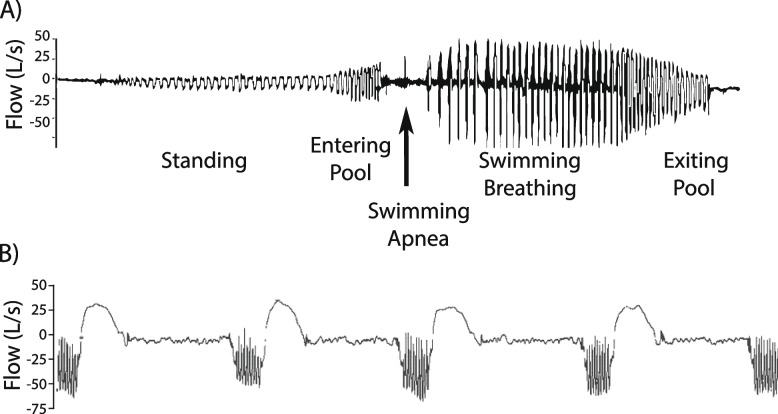
Inspiratory and expiratory air flow (L/s) measurements taken with the ergospirometry mask system. Positive value is inspiratory flow, negative is expiratory flow. **A** Airflow signal (L/s) shown over an entire length of free swimming (75 m effective). **B** Air flow signal (L/s) over five consecutive breaths during free swimming. Note the marked oscillations during the expiratory period

**Fig. 3:**
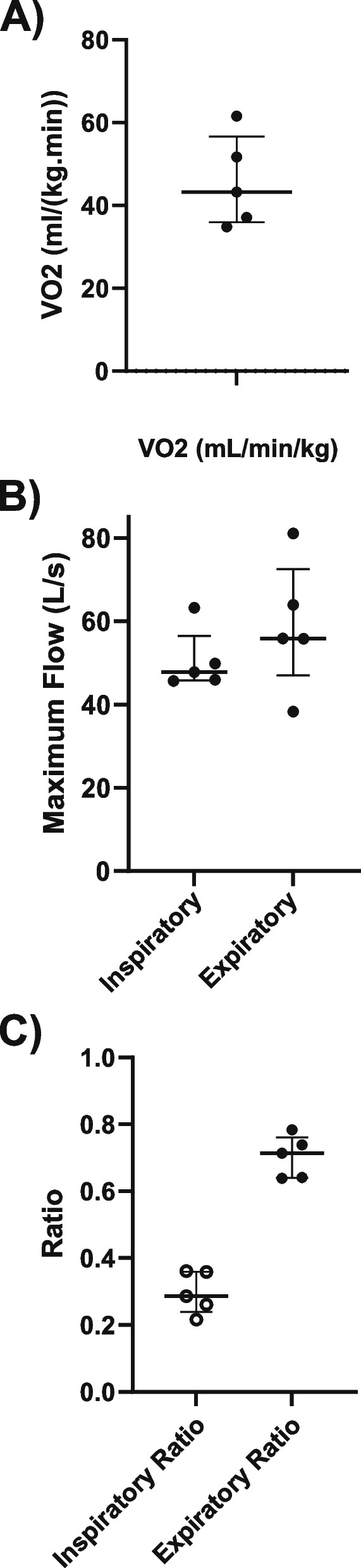
Oxygen consumption in ml/(kg/min) (**A**), maximum inspiratory and expiratory flow in L (**B**), and inspiratory and expiratory ratios (**C**) for five horses during straight-line untethered free swimming

Ventilation was not consistent throughout the entire swimming period, as illustrated in Fig. 2A. Upon entering the pool, there was a period of apnea of approximately 20–30 s, followed by approximately 30 s of bradypnea at a median of 12 breaths/min (10–16). This period of bradypnea was followed by regular breathing at an average Rf of 31.4 breaths/min (20.0–33.8) (Table 1). The median V̇O_2_ during swimming was 43.2 ml/(kg/min) (36.0–56.6) (Fig. 3A). The peak inspiratory (PkV_I_) and expiratory (PkV_E_) flow rates were 47.8 L/s (45.8–56.5) and 55.8 L/s (38.3–72.5), respectively (Fig. 3B). The expiratory flow tracings showed marked oscillations that coincided with a vibrating expiratory sound (Fig. 2B). The inspiratory volume (V_I_) was slightly greater than the expiratory volume (V_E_), with the expiratory time (t_E_) being more than double the inspiratory time (t_I_) (Table 1), resulting in an inspiratory ratio (t_I_:t_tot_) of 0.3 and an expiratory ratio (t_E_:t_tot_) of 0.7 (Fig. 3C).

**Table 1:** Spirometry, cardiovascular, and biochemical parameters of five horses undergoing free swimming exercise (1 × 75 m effective length, straight swimming). Ventilation and heart rate were measured continuously during swimming. Blood was drawn immediately after the swimming exercise and blood lactate and ammonia concentrations measured. Values for each horse and overall median and interquartile values are listed

Horse ID	Respiratory frequency (breath/min)	Peak inspiratory volume (L)	Peak expiratory volume (L)	Minute ventilation (L/min)	Inspiratory period (s)	Expiratory period (s)	Average heart rate (bpm)	Max heart rate (bpm)	Blood lactate (mmol/L)	Blood ammonia (µmol/L)
**1**	12.8	21.7	19.0	279.3	0.6	1.0	178	216	1.8	41.0
**2**	31.8	21.8	18.9	693.0	0.7	1.2	185	214	2.0	43.0
**3**	31.3	16.7	10.8	522.9	0.5	1.4	157	188	1.5	36.0
**4**	35.7	16.2	14.0	580.0	0.5	1.2	179	208	1.1	37.0
**5**	27.1	15.3	14.7	414.7	0.5	1.7	140	157	0.8	44.0
	31.4 (20.0–33.8)	16.7 (15.3–21.8)	14.7 (12.4–18.9)	522.9 (414.7–580.0)	0.5 (0.5–0.6)	1.2 (1.1–1.6)	178.0 (148.5–182.0)	208.0 (172.5–215.0)	1.5 (1.0–1.9)	41.0 (36.5–43.5)

### Heart rate

HR was observed to peak during entry (walking down the ramp) into the pool, with a maximum HR of 208 bpm (172.5–215.0). Once the horses began swimming, the HR gradually decreased throughout the length of the swim to an average of 178.0 bpm (148.5–182.0) (Table 1).

### Blood lactate and ammonia levels

Blood lactate and ammonia concentrations measured immediately following free swimming were low, with blood lactate concentrations well below 4 mmol/L (Table 1).

## Discussion

This study was technically challenging to conduct and represents a significant contribution to the understanding of swimming exercise and the respiratory response of horses to it. Research in this area is scarce, primarily due to technological limitations imposed by recording physiological parameters in or under water. To partly overcome these limitations, horses were recruited for the present study based on their natural swimming technique and high head carriage to minimize the risk of the facemask becoming submerged. Even with this recruitment approach, one instance of a horse submerging the mask occurred, resulting in data loss. An outward and upward extension of the pneumotachographs, similar to a snorkel, was tested in a pilot study prior to data collection; however, these extensions were found to be too heavy and cumbersome during swimming and were not used.

In as early as 1976, swimming was documented to be beneficial in the rehabilitation and conditioning of racehorses [1]. In a 2019 survey, 38.1% of all polled Australian Standardbred racehorse trainers and 60% of leading trainers indicated that they use swim exercise to rehabilitate, condition, and provide mental stimulation to horses [2]. However, approximately 16% of all trainers stated that they did not use swimming as part of their training routine due to a lack of beneficial effects. Interestingly, surveyed trainers reported that horses swam for an average duration of 10 min [2], while Murakami et al*.* reported that horses tolerated 60 min of swimming exercise [1]. The frequency and duration of swimming exercise employed varies widely, perhaps explaining the variable perception of the efficacy of this form of exercise in racehorse development [2]. Data on the physiological responses of horses to swim exercise are scarce, making it difficult for trainers to effectively integrate swimming into standard conditioning and rehabilitation programs.

This study indicated that free swimming represents a primarily aerobic, submaximal exercise in horses. During free swimming, horses adopted a unique breathing pattern, with a relatively longer apneic period at the beginning of the exercise and an inspiration time less than half that of expiration, which was consistent with what has previously been reported for swimming in horses [3, 5]. The apneic period was longest at the beginning of swimming exercise, perhaps in an attempt to protect the airway from water, increase buoyancy, or as part of the MDR [3]. However, unlike the typical manifestation of the MDR, no bradycardia was observed [21]. It has previously been suggested that swimming can be a stressful event in horses, with hesitancy or reluctance to enter the water being noted, which may explain this prolonged period of apnea [3, 15, 17, 22]. Furthermore, we have previously reported an anticipatory response to swimming in horses, with HR being elevated during entry into the water and initial swimming [17]. Interestingly, in humans, apnea has been associated with arrhythmias; however, this association has not been observed in swimming horses [17]. If the apneic period is associated with an anticipatory or stress response, modifications to the water temperature, swim entry area/ramp or greater habituation may modulate this response.

It has previously been reported that the apneic period coincides with flattening or near closure of the nares, closure of the *rima glottidis*, and collapse of the nasopharynx [3]. We observed a clear collapse of the nostrils during swimming, likely to minimize entry of water into the airways. A reduction in Rf has previously been reported in aquatic and semiaquatic animals when they are resting at the surface of water [23]. We observed bradypnea for the first 30 s of swimming (12 breaths/min), with Rf approximately doubling (to a median of 31 breaths/min) for the remainder of the swim length to values consistent with what has been reported for tethered swimming (~ 24–48 breaths/min) [3, 5, 13, 14, 20, 24] and water treadmill walking (~ 46 breaths/min) [25]. Respiratory frequencies of approximately 20 to 50 breaths/min are significantly lower than what has been reported in horses walking on a standard, dry treadmill (~ 65–80 breaths/min) [26, 27]. Similar to the report by Hobo et al. [5], we observed that the length of the expiratory period was more than double the length of the inspiratory period, resulting in a t_I_/t_tot_ ratio of 0.3. This finding is in contrast to what Jones et al. reported, where expiration was short and “explosive” [3]. In fact, peak expiratory pressure has been reported to be more than double during swimming compared to galloping in horses [14]. The breathing strategy during swimming is different from that during water treadmill exercise, where t_E_ and t_I_ are more similar in duration (0.9 s and 0.8 s, respectively), with t_E_ being shorter than what was observed in the present study [25]. Compared to those of galloping overground at submaximal exercise intensities, the peak flows (~ 50 L/sec) and tidal volume (~ 15 L) values were relatively comparable, although the VE was much lower (~ 523 L/min) during swimming [28, 29]; this was mostly due to the lower respiratory frequency observed while swimming and the period of apparent breath holding when there was no airflow. The findings of the current study reaffirm that the way in which horses breathe when swimming is very different from the pattern they exhibit when cantering or galloping. Whereas breathing at these gaits has two constant switching phases—inspired and expired—with no notable pause in airflow, there were clearly three phases related to the breathing pattern of our swimming horses—inspired, breath-holding or apnea—which was characterized by zero airflow in or out and expiration. During tethered swimming exercise, complete upper airway collapse was observed during the apneic period in all horses, which the authors suggested may aid in buoyancy or be associated with MDR [3]. The flow tracings and the maximal flow values in the present study suggest that the upper airways of these horses were not obstructed when air was flowing into or out of the lungs during free swimming breaths. However, it is possible that the airways were still intermittently collapsing during the apneic period when we observed zero airflow and closure of the nostrils. The only way to verify this possibility would be to endoscopically visualize the upper respiratory tract while simultaneously measuring airflow, which was not technologically possible in this study. Although the spirometry data suggest that the upper airways were fully functional when air was flowing during the horses’ breathing efforts, this does not mean that they did not actively narrow secondary to contraction of the pharyngeal muscles at the same time that the nostrils closed. It may also be that there are differences in the way horses swim depending on whether they are tethered or untethered, with free swimming requiring less work effort and potentially being a less stressful activity than tethered swimming. This possibility has not been specifically investigated, and the same can be said of evaluating possible breed differences in the way horses breathe while swimming—i.e., Arabian endurance horses versus Standardbred and Thoroughbred racehorses.

Free swimming appears to be a lower intensity exercise than tethered swimming, with the V̇O_2_ observed here [~ 43 ml/(kg/min)] being within the lower range of what has previously been reported during tethered swimming [4]. In that study, horses were tethered in place via a tailrope and encouraged to swim at increasingly greater workloads. Of the four horses used in that study, three had a V̇O_2_ less than 60 ml/(kg/min) when they swam tethered at self-selected speeds (i.e., without further encouragement). The maximal V̇O_2_ values that were obtained were less than 120 ml/(kg.min) for all the horses. The average V̇O_2_ obtained in the present study corresponds to a work effort between 3 and 4 (kg pulled.kg body weight/100) [4]. In our study, horses were habituated to swimming exercise and were permitted to swim at a self-selected speed without additional resistance or encouragement from handlers. Perhaps with encouragement to swim at higher speeds, similar workloads could be achieved during free swimming. However, if the intent is to achieve the greatest possible workload, tethered swimming may be more appropriate than free swimming. Previously reported work involving walking on a water treadmill at stifle depth water achieved V̇O_2_ values up to 16.7 ml/(kg/min) [25], nearly half of what we observed during free swimming, and approximately one-third of what has been reported for tethered swimming, indicating that swimming is more strenuous than water treadmill exercise, even in deep water.

Unlike V̇O_2_, HR values do not appear to differ between free and tethered swimming, ranging between 117 and 220 bpm during free swimming [5, 7, 8, 13, 16, 17, 19, 22, 24, 30] and 100 to 216 bpm during tethered swimming [3, 4, 14, 20] between studies under various conditions. In another study of Arabian endurance horses, we reported an average swimming HR of 162 bpm [17], which is very similar to what we observed in the present study (178 bpm). The HR values obtained in our study are similar to those obtained by Nicholl et al*.* when horses were tethered at a work effort of 8 (kg pulled.kg body weight/100). Interestingly, this work effort was found to result in a V̇O_2_ of approximately 100 ml/(kg/min) [15], nearly double what we observed. Regardless of swimming type (free versus tethered), HR values obtained during swimming are lower than those previously reported for galloping thoroughbreds (~ 215 to 240 bpm) [31] and trotting standardbreds (~ 216 bpm) [32], indicating that swimming represents a lower workload. The free-swimming HR values are significantly greater than what we previously reported during walking on a water treadmill (~ 58 bpm) [25], again indicating that free swimming poses a significantly greater workload than slow-water treadmill exercise. We previously reported that cardiac arrhythmias were observed in 27% of swimming ECGs, a majority of which were clinically irrelevant [17]. However, quantifying the presence and types of arrhythmias was beyond the scope of the present study.

We found that blood lactate and ammonia levels were low following this swimming exercise. Free and tethered swimming exercise reportedly results in lactate levels less than 11 mmol/L [3–5, 7–9, 13, 19, 22]. An average blood lactate concentration of 2.3 mmol/L was previously reported following two 60-m laps of free swimming. The present study included only a single 75 m effective swimming length, which likely explains the reduced lactate level reported here. Previously reported values of blood lactate (< 1 mmol/L) are lower following water treadmill exercise [25, 33, 34], with similar lactate levels (1.5–2 mmol/L) being obtained at greater speeds during water treadmill exercise [35]. An average blood lactate level of 1.5 mmol/L was significantly lower than the hypothetical anaerobic threshold of 4 mmol/L, confirming that the swimming exercise employed in this study was submaximal.

This study has several limitations. The study was limited to a very small number of horses and was limited to one breed, discipline, and competition level. Due to technological limitations, data were collected only from horses with a specific swimming style and head carriage. Furthermore, the data were collected only from horses that were swimming in a straight line and untethered. Therefore, further research on a greater variety of horses and swimming types is warranted.

## Conclusion

This study confirmed that free swimming is an aerobic, submaximal form of exercise that is not a suitable replacement for high-intensity work, such as galloping. The workload associated with free swimming is less than that associated with tethered swimming but is greater than that associated with water treadmill exercise. However, free swimming may be integrated into standard training programs to replace lower-intensity exercise to some degree, thereby reducing the wear and tear on the limbs that is associated with over-ground locomotion. Further work to understand the potential long-term effects of prolonged swimming exercise on respiratory function and fitness is warranted.

## Methods

The study was approved by the Institutional Animal Care and Use Committee of the Equine Veterinary Medical Center, a member of the Qatar Foundation (EVMC-2020–1135), and was performed at the Al Shaqab Equine Exercise Center. The horses were owned by the Al Shaqab Equine Center, and informed consent was obtained from the trainers to enroll the horses in the study.

### Animals

Five healthy, elite endurance horses were enrolled in the study (all Arabian horses; two mares, three geldings; mean age ± standard deviation (SD), 13.3 ± 2.2 years; mean weight ± SD, 430.0 ± 26.7 kg). Horses were actively enrolled in an elite endurance training program and confirmed to be healthy after undergoing a clinical examination. The horses had no history of recent lameness, health issues, or poor performance. All the horses were familiar with the pool and had previously undergone a minimum of two months of training that included regular swimming sessions in the same pool utilized for data collection. Horses were observed swimming prior to beginning the study to select horses that naturally swam with a high head carriage to allow for the use of the ergospirometry facemask equipment.

### Experimental protocol

The data were collected in a large (100 m long, 3 m deep), straight pool. Horses swam untethered and were restrained by a halter and loose lead ropes by two handlers positioned on each side of the pool. Handlers were instructed to allow the horse to swim at individual preferred speeds without encouragement. The data were collected for a single 75 m swimming length. The water temperature was maintained at 31ºC for all the data collection.

### Oxygen consumption and ventilation

Horses were equipped with a portable ergospirometry facemask (Department of Veterinary Clinical Sciences, Pullman, USA) to measure V̇O_2_ and ventilatory parameters (V_I_, V_E_, PkV_I_, PkV_E_, Rf, VE, t_I_, t_E_, t_I_/t_tot_, t_E_/t_tot_) [36]. Prior to data collection, the horses were habituated to the facemask overground and during swimming (the “shell” facemask was used only without a flowmeter or other sensors) until they could calmly wear it. Horses were videotaped while swimming without the facemask to establish standard breathing patterns (respiratory frequency and periods of apnea). Horses were judged to be habituated to swimming with the facemask shell system when their breathing pattern with the facemask matched that without the facemask.

The facemask system (flowmeter and gas analyzer) was calibrated according to the manufacturer’s instructions using room air and 16% O_2_ prior to data collection for each horse. The facemask was installed on the horse and connected to a gas analysis and data acquisition system that was placed in a backpack and suspended above the water beside the horse while it was swimming (Fig. 1). Internal padding was added for each horse to minimize dead space within the facemask. Ventilatory results were calculated using the manufacturer-supplied software. Periods of apnea were reflected and timed by observing prolonged periods of zero (0 L/s) airflow. Environmental condition data (ambient temperature, barometric pressure, humidity) and horse weight were input for analysis. The results are reported as standard temperature and pressure (STPD) values. Breath-by-breath recordings were analyzed manually for the entirety of the swimming length. Only data that had clear V̇O_2_ and flow were used for analysis.

### Heart rate

Horses were instrumented with a telemetric ECG device (TELEVET 100, Engel Engineering Service, software version 7.0.0) for continuous HR measurements. Four adhesive electrodes (Skintact, Leonhard Lang) were positioned in a modified base-apex configuration. The electrodes were covered with an additional adhesive foam pad that prevented water entry, as previously described [17]. The ECG recording equipment was placed in a waterproof pouch that was attached to the crown piece of the halter and maintained above the surface of the water. ECG recordings were manually inspected, and the maximum HR and average HR (over the entire swim length) were calculated.

### Blood lactate and ammonia levels

Blood samples (< 5 ml) were collected in vacutainer tubes containing potassium oxalate from the jugular vein immediately following exercise. A handheld lactate analyzer (Lactate Scout + , EKF Diagnostics) was used to immediately measure the blood lactate concentration. The calibration was checked prior to data collection as per the manufacturer’s recommendations. A handheld ammonia analyzer (PocketChem BA, Arkray, Inc.) was used to immediately measure the blood ammonia concentration, and calibration was also checked prior to data collection as per the manufacturer’s recommendations.

### Analysis

Descriptive analysis of all the measured outcomes (V̇O_2_, V_I_, V_E_, PkV_I_, PkV_E_, Rf, VE, t_I_, t_E_, t_I_/t_tot_, t_E_/t_tot_, HR, lactate, and ammonia) were performed. All the data are reported as medians with interquartile ranges.

## Data Availability

The datasets used and/or analyzed during the current study are available from the corresponding author on reasonable request.

## Abbreviations

ECG: Electrocardiogram
HR: Heart rate
Min: Minute(s)
MDR: Mammalian dive reflex
PkV_E_: Peak expiratory flow
PkV_I_: Peak inspiratory flow
Rf: Respiratory frequency
S: Second(s)
STPD: Standard temperature and pressure, dry
t_E_: Expiratory period
t_I_: Inspiratory period
t_E_/t_Tot_: Expiratory ratio
t_I_/t_Tot_: Inspiratory ratio
VE: Minute ventilation
V_E_: Expired volume
V_I_: Inspired volume
V̇O_2_: Oxygen consumption
